# Meaningful Engagement of Residents With Dementia in Assisted Living: The Role of Direct Care Workers

**DOI:** 10.1093/geroni/igab046.980

**Published:** 2021-12-17

**Authors:** Jennifer Craft Morgan, Joy Ciofi, Candace Kemp, Andrea Hill, Alexis Bender

**Affiliations:** 1 Georgia State University, Georgia State University, Georgia, United States; 2 Georgia State University, Avondale Estates, Georgia, United States; 3 Gerontology Institute, Georgia State University, Atlanta, Georgia, United States; 4 Georgia State University, Atlanta, Georgia, United States; 5 Emory University, Atlanta, Georgia, United States

## Abstract

Meaningful engagement has important implications for quality of care for persons living with dementia. Yet, little research has focused on direct care workers’ (DCWs) role in facilitating engagement opportunities for residents in assisted living. Using data from our ongoing NIA-funded study, “Meaningful Engagement and Quality of Life among Assisted Living Residents with Dementia,” we describe DCW approaches to engaging residents and the factors that influence the use and successful application of these approaches. Focal residents (N=33) were followed at four diverse assisted living communities for one year. Data includes care partner interviews (N=100), including 28 DCWs and 1560 hours of field observation data. DCWs interviewed had between 2 months and 12 years’ experience in their current position and were mostly African American and/or immigrant women of color. Findings suggest that DCW-resident interactions are key opportunities to engage residents in a meaningful way and can facilitate positive, trust-based relationships. This analysis elaborates on our previous work identifying four approaches: knowing the person, connecting with and meeting people where they are, being in the moment, and viewing all encounters as opportunities. We identified factors affecting opportunities for, and experiences with, meaningful engagement between residents and DCWs, including community staffing, consistent assignment, scheduling, DCW training and tenure, and community resources. We conclude with implications for practice emphasizing how elaboration of these approaches can inform the development of DCW training, opportunities for career advancement, and integration of approaches consistently into daily practice in an effort to support meaningful engagement of residents living with dementia.

